# FAM19A5 Expression During Embryogenesis and in the Adult Traumatic Brain of *FAM19A5-LacZ* Knock-in Mice

**DOI:** 10.3389/fnins.2019.00917

**Published:** 2019-08-30

**Authors:** Anu Shahapal, Eun Bee Cho, Hyo Jeong Yong, Inyoung Jeong, Hoyun Kwak, Jae Keun Lee, Wonkyum Kim, Bongcheol Kim, Hae-Chul Park, Won Suk Lee, Hyun Kim, Jong-Ik Hwang, Jae Young Seong

**Affiliations:** ^1^Graduate School of Biomedical Sciences, Korea University College of Medicine, Seoul, South Korea; ^2^Neuracle Science Co., Ltd., Seoul, South Korea; ^3^Graduate School of Biomedical Sciences, Korea University Ansan Hospital, Ansan, South Korea

**Keywords:** FAM19A5, traumatic brain injury, brain development, neuron, oligodendrocyte precursor cells

## Abstract

FAM19A5 is a secretory protein that is predominantly expressed in the brain. Although the *FAM19A5* gene has been found to be associated with neurological and/or psychiatric diseases, only limited information is available on its function in the brain. Using *FAM19A5-LacZ* knock-in mice, we determined the expression pattern of FAM19A5 in developing and adult brains and identified cell types that express FAM19A5 in naïve and traumatic brain injury (TBI)–induced brains. According to X-gal staining results, FAM19A5 is expressed in the ventricular zone and ganglionic eminence at a very early stage of brain development, suggesting its functions are related to the generation of neural stem cells and oligodendrocyte precursor cells (OPCs). In the later stages of developing embryos and in adult mice, FAM19A5 expression expanded broadly to particular regions of the brain, including layers 2/3 and 5 of the cortex, cornu amonis (CA) region of the hippocampus, and the corpus callosum. X-gal staining combined with immunostaining for a variety of cell-type markers revealed that FAM19A5 is expressed in many different cell types, including neurons, OPCs, astrocytes, and microglia; however, only some populations of these cell types produce FAM19A5. In a subpopulation of neuronal cells, TBI led to increased X-gal staining that extended to the nucleus, marked by slightly condensed content and increased heterochromatin formation along the nuclear border. Similarly, nuclear extension of X-gal staining occurred in a subpopulation of OPCs in the corpus callosum of the TBI-induced brain. Together, these results suggest that FAM19A5 plays a role in nervous system development from an early stage and increases its expression in response to pathological conditions in subsets of neurons and OPCs of the adult brain.

## Introduction

FAM19A5, also called TAFA5, is a member of the TAFA family of secreted proteins that are predominantly expressed in the brain (Tom Tang et al., 2004). Due to the presence of conserved CC motifs, this family has been considered as an atypical member of the CC-chemokine family (Tom Tang et al., 2004). In addition, FAM19A5 is also regarded as a putative neuropeptide because it is co-localized with vasopressin and oxytocin in magnocellular and parvocellular neurons of the hypothalamic paraventricular nucleus, which are involved in fluid homeostasis (Paulsen et al., 2008). FAM19A5 expression in the mouse brain was found to increase at the later stages of embryonic development (Yue et al., 2014), suggesting a role of FAM19A5 in brain development. Genome-wide association studies have demonstrated an association of *FAM19A5* with late-onset Alzheimer disease in humans (Herold et al., 2016; Mez et al., 2017). Mosaic monosomy of chromosome 22—which includes disruption of the *FAM19A5* gene—leads to skeletal abnormalities, low body weight, and neuropsychiatric problems, including attention deficit hyperactivity disorder (ADHD), aggression, or autistic symptoms (Kashevarova et al., 2018). Multiple gene copies of the *FAM19A5* gene seems to be associated with glioma in some patients (Díaz De Ståhl et al., 2005), implying the role of FAM19A5 in tumorigenesis of the central nervous system (CNS). These observations indicate that FAM19A5 has roles in neural development and the pathological conditions of neurological and/or psychiatric diseases.

In addition, FAM19A5 may have functions at the peripheral tissues. For instance, FAM19A5 likely inhibits the RANKL-induced differentiation of osteoclast precursor cells by interacting with formyl peptide receptor 2 (Park et al., 2017). A recent report showed that FAM19A5 is secreted from adipose tissues and inhibits the proliferation and migration of smooth muscle cells. In particular, FAM19A5 was found to suppress neointima formation in injured rat carotid arteries by interacting with sphingosine-1-phosphate receptor 2 (Wang et al., 2018).

Since the first report on FAM19A5 expression in the brain (Tom Tang et al., 2004), only limited information is available on the function of FAM19A5 in the brain and peripheral tissues. In this study, using *FAM19A5-LacZ* knock-in (KI) mice, we determined the expression pattern of FAM19A5 during embryogenesis and in the adult brain, and identified cell types in the brain that express FAM19A5. Traumatic brain injury (TBI) is a sudden insult to the brain from an external force which may result in permanent or temporal brain dysfunctions. Various cellular mechanisms are activated after TBI to regenerate or replace the damaged or dead cells. Some genes important in development reactivate under injury condition (Mierzwa et al., 2014; Chaboub et al., 2016). Therefore, we analyzed changes in FAM19A5 expression in response to TBI.

## Materials and Methods

### Generation of *FAM19A5-LacZ* Knock-in Mice

**FIGURE 1:**
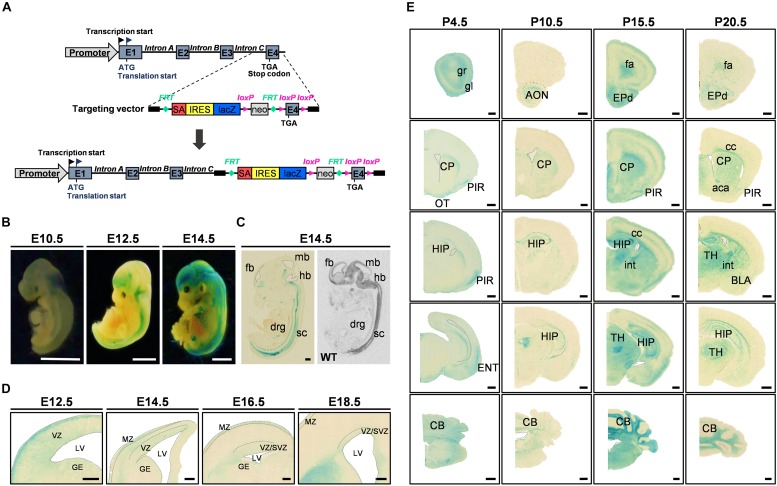
*FAM19A5-LacZ* knock-in (KI) gene construct and FAM19A5 expression in the nervous system at developmental stages. **(A)** Schematic diagram of the *FAM19A5-LacZ* KI mouse gene construct. The target vector containing the *LacZ* gene is inserted in front of exon 4 (E4) of the *FAM19A5* gene by homologous recombination, producing β-galactosidase enzyme under control of the *FAM19A5* gene promoter. **(B)** Whole embryo X-gal staining at embryonic days E10.5, E12.5 and E14.5 of *FAM19A5-LacZ* KI homozygote (+/+) mice. X-gal staining occurred from the early embryonic stage E10.5, with increased and continuous staining in the later embryonic stages. **(C)** Comparison of sagittal views between X-gal staining (left) and *in situ* hybridization pattern (right) at E14.5. Mouse and rat embryos at E14.5 were subjected to X-gal staining and *in situ* hybridization, respectively, revealing a similar pattern of expression. **(D)** FAM19A5 expression in the germinal zones; ventricular zone (VZ), subventricular zone (SVZ) and ganglionic eminence (GE). Coronal view of E12.5, E14.5, E16.5, and E18.5 X-gal–stained sections. **(E)** X-gal staining for coronal brain sections at postnatal stages P4.5, P10.5, P15.5, and P20.5, suggesting broad and continuous FAM19A5 expression throughout the postnatal stages. aca, anterior commissure, anterior part; AON, anterior olfactory nucleus; BLA, basolateral amygdaloid nucleus, anterior part; CB, cerebellum; cc, corpus callosum; CP, caudate putamen; drg, dorsal root ganglion; ENT, entorhinal cortex; EPd, dorsal endopiriform nucleus; E1, exon 1; E2, exon 2; E3, exon 3; E4, exon 4; fa, forceps minor of the corpus callosum; fb, fore brain; GE, ganglionic eminence; gl, glomerular layer of the olfactory bulb; gr, granular layer of the olfactory bulb; hb, hind brain; HIP, hippocampal region; int, internal capsule; IRES, internal ribosome entry site; LV, lateral ventricle; mb, mid brain; MZ, marginal zone; neo, neomycin phosphotransferase gene; OT, olfactory tubercle; PIR, piriform cortex; SA, splice acceptor site; sc, spinal cord; SVZ, subventricular zone; TH, thalamus; VZ, ventricular zone; WT, wild-type. Ventricles (lateral ventricle, 3rd ventricle and 4th ventricle) are demarcated by dashed lines in panel C-E. All animals used were *FAM19A5-LacZ* KI mouse model otherwise labeled. Scale bars represent 2 mm for panel B, 500 μm for panels C and E, and 100 μm for panel **D**.

*FAM19A5-LacZ* KI mice were generated by the UC Davis Mouse Biology Program. The *FAM19A5*-targeting vector was constructed as shown in Figure 1A. The gene-trap method using *LacZ* as a reporter gene was employed to visualize FAM19A5 expression in tissue sections (Mountford et al., 1994). Briefly, the target vector containing IRES-*lacZ* gene was inserted in front of exon 4 of the *FAM19A5* gene. The *LacZ* gene is expressed independently of the target *FAM19A5* gene due to the IRES element. This *FAM19A5*-targeting vector was delivered to embryonic stem cells by electroporation. We confirmed the incorporation of this vector into the target chromosome by genotyping and chromosome counting of transgenic embryonic stem cells. Selected transgenic embryonic stem cells were injected into blastocysts, and the embryos were implanted into the uterus of female recipient mice. We performed a germline transmission test to check for stable germline expression in the chimeric generation. The following primers were used for genotyping: FAM19A5-F1, 5′-TGG TCA GAA CTG TGT GAG TGC-3′; FAM19A5-R1, 5′-CAC CAT GGG CAA GTT TAA CA-3′; and FAM19A5-R2, 5′-CCA ACC CCT TCC TCC TAC AT-3′ (Supplementary Figures 1A,B). The generated *FAM19A5-LacZ* KI chimeric mice were backcrossed onto C57BL/6J genetic background. Wild-type C57BL/6J female mice were purchased from Orient Bio, Inc. (Seongnam, South Korea) and mated with heterozygous KI males. To obtain homozygous *FAM19A5-LacZ* KI mice, the heterozygous male mice were mated with the heterozygous female mice. Their wild-type littermates produced by the backcrossing were used for the control groups.

### Animals

Mice were housed in temperature-controlled (22–23°C) conditions with a 12-h light/12-h dark light cycle (lights on at 8:00 am). The mice were given *ad libitum* supplies of standard chow and water. All animal experiments were designed to use the fewest mice possible, and anesthesia was administered. All animal procedures were approved by the Institutional Animal Care and Use Committee of Korea University (KOREA-2016-0091-C3).

### Traumatic Brain Injury

8-to-9-week-old *FAM19A5-lacZ* KI mice were anesthetized with sodium pentobarbital (50 mg/kg). Cryogenic TBI was performed by placing a prechilled iron rod on the calvarium for 1 min (Moon et al., 2011). Animals were sacrificed at 7 days post-injury.

### X-gal Staining for Embryo, Postnatal, and Adult Brains

For embryonic X-gal staining, the pregnant mice were sacrificed by cervical dislocation, and the embryos were isolated. Whole embryos at embryonic day 10.5 (E10.5), E12.5, and E14.5 were fixed in 4% paraformaldehyde (PFA) and 0.2% glutaraldehyde (GTA) in phosphate buffer (PB) at 4°C for 10, 15, and 30 min, respectively. For embryos older than E14.5, the heads were cut and the skins were removed. The heads of the embryos were fixed in the same fixative for 1∼2 h at 4°C. For postnatal mice, the brains were isolated from the skulls and fixed in the same fixative for 1∼2 h at 4°C. The fixed tissues were then washed with phosphate-buffered saline (PBS) twice for 5 min and incubated in X-gal staining solution, 1 mg/ml of X-gal, 2 mM MgCl_2_, 5 mM EGTA, 5 mM potassium ferrocyanide, 5 mM potassium ferricyanide, 0.01% sodium deoxycholate, and 0.02% Non-idet-P40 in 0.1 M PB at pH 7.4 for 48 h at 37°C in the dark. The stained tissues were post-fixed with 4% PFA in PBS overnight at 4°C and washed, then whole brain images were obtained.

For X-gal–stained sections from E12.5 to postnatal day (P4.5), the stained whole brains were cryoprotected with 30% sucrose in PBS and sectioned at 40 μm using a cryostat (Leica, Wetzlar, Germany). For X-gal–stained sections from P10.5, P15.5, P20.5, and 10-week old adult male mice, animals were perfused with 4% PFA and 0.2% GTA in PB. The brains were isolated and post-fixed in 0.2% GTA in PB for 24 h at 4°C. The brains were then cryoprotected in 30% sucrose in PBS and serially cross-sectioned in 40 μm using the cryostat. The sectioned tissues were then incubated in X-gal staining solution for 48 h at 37°C in the dark. The images of the sections were taken using a slide scanner (Axio scan Z1, Zeiss).

### Immunofluorescence Analysis of Brain Sections

For X-gal staining with multiple fluorescence labeling, 10-week old adult male mice were perfused with 4% PFA in PBS, and isolated brains were post-fixed in the same solution for 3 h. Brains were then cryoprotected in 30% sucrose in PBS, serially sectioned with the cryostat (20 μm slices), and stored in 50% glycerol in PBS at −20°C. For X-gal staining, sections were brought to room temperature and washed three times in PBS for 5 min each, then transferred to X-gal staining solution at 37°C overnight. After X-gal staining, sections were blocked with 3% bovine serum albumin and 0.1% Triton X-100 in PBS for 30 min and incubated in primary antibodies overnight at 4°C. The primary antibodies used in this study were mouse anti-NeuN (1:1000, Millipore), rabbit anti-NeuN (1:500, Millipore), rabbit anti-GFAP (1:1000, Wako), rabbit anti-NG2 (1:500, Millipore), rabbit anti-Iba1 (1:500, Dako), sheep anti-Sez6l2 (1:500, R&D Systems), rabbit anti-Tuj1 (1:1000, SIGMA), mouse anti-nestin (1:500, Millipore), rat anti-CD31 (1:500, BD Biosciences), rabbit anti-PDGFRβ (1:500, Abcam), goat anti-CD45 (1:500, R&D Systems), rabbit anti-MBP (1:500, Abcam), rabbit anti-MAP2 (1:500, Millipore), rabbit anti-Olig2 (1:500, Millipore), rabbit anti-Ki67 (1:500, Abcam), rabbit anti-Active caspase-3 (1:500, Cell signaling), and mouse anti-O4 (1:500, Millipore). Following several PBS washes, the appropriate secondary antibody was applied for 30 min. Nuclei were labeled with Hoechst 33342 (Invitrogen, Carlsbad, CA, United States). For the fluorescent TUNEL assay, co-labeled sections were stained using an *in situ* cell death detection kit (Roche) according to the manufacturer’s instructions. The sections were washed, mounted, and imaged using a confocal microscope (TCS SP8, Leica) as previously described (Levitsky et al., 2013). Briefly, the X-gal–stained sections were excited at 633 nm, and the fluorescence signals emitted at 650–770 nm were visualized. Differential interference contrast microscopy images were also obtained to confirm real X-gal fluorescence precipitates. Confocal acquisition of the additional fluorescence labels was conducted as follows: Hoechst (excited at 405 nm and detected between 415–450 nm) for the nucleus; Alexa 488 (excited at 488 nm and detected between 489– 550 nm) and Alexa 555 (excited at 561 nm and detected between 563 and 620 nm) for other cell type–specific markers. For the quantification of nuclear and cytoplasmic X-gal fluorescence, Z-stack images of between 10 and 15 μm in depth were acquired at 1 μm intervals using a 20 X objective.

### Immunoenzyme (HRP) Analysis of Brain Sections

For co-staining of X-gal and 3, 3′-diaminobenzidine (DAB) staining, the X-gal–stained brain sections were incubated in 0.3% H_2_O_2_ solution for 10 min and washed in PBS for 5 min at room temperature. Sections were blocked with 3% bovine serum albumin and 0.1% Triton X-100 in PBS for 30 min and incubated in primary antibodies overnight at 4°C. The primary antibodies used in this study were mouse anti-NeuN (1:1000, Millipore), rabbit anti-GFAP (1:1000, Wako), rabbit anti-NG2 (1:500, Millipore), and rabbit anti-Iba1 (1:500, Dako). Following several PBS washes, the appropriate biotinylated secondary antibody was applied for 30 min. The sections were subjected to the avidin-horseradish peroxidase complex method (Vector Laboratories, Burlingame, CA) for 30 min at room temperature and finally treated with DAB (SIGMA) until desired dark brown color was generated. The images of the sections were taken using a slide scanner (Axio scan Z1, Zeiss).

### Nissl Staining

For Nissl staining following the X-gal staining, the sections were placed in PBS for 5 min each, then hydrated in 1% Cresyl violet at 50°C for 20 min. The sections were rinsed with distilled water, dehydrated, and mounted with Permount (Thermo Fisher Scientific). The slide was imaged using an optical microscope.

### *In situ* Hybridization

Adult mice and rats were sacrificed, and mouse brains and embryonic rats were removed and quickly frozen in isopentane on dry ice. Tissue sections were cut to 20 μm thickness with a cryostat, thaw-mounted on Superfrost Plus slides (Thermo Fisher Scientific, United States), and stored at −70°C until use. Sections were fixed in 4% PFA, washed with PBS, and acetylated with 0.25% acetic anhydride in 0.1 M triethanolamine/0.9% NaCl (pH 8.0). Samples were hybridized overnight with a radiolabeled probe (1.2 × 106 cpm) and washed four times with 2 × standard sodium citrate (SSC). A template for the FAM19A5 probe was prepared by subcloning the RT-PCR products into a pGEM-T vector (Promega). For the preparation of a radiolabeled mouse FAM19A5 cDNA probe, the following primers were used: mFAM19A5-F, 5′-ATG CAG CTC CTG AAG GCG CT-3′; mFAM19A5-R, 5′-TCA GGA GAC CGT GGT GGT CT-3′. For the preparation of a radiolabeled rat FAM19A5 cDNA probe, the following primers were used: rFAM19A5-F, 5′-ATG CAG CTC CTG AAG GCG CTC-3′; rFAM19A5-R, 5′-TCA GGA GAC CGT GGT GGT CT-3′.

Sense and antisense riboprobes were prepared using an *in vitro* transcription system (Promega) in the presence of [α-^35^S] UTP (Amersham Pharmacia Biotech, United States). After RNase A treatment, slides were rinsed with 2×, 1×, 0.5×, and 0.1× standard sodium citrate containing 1 mM dithiothreitol for 10 min each at room temperature, then washed with 0.1× standard sodium citrate at 60°C. The samples were dehydrated in ethanol and exposed to X-ray film (Biomax MR, Kodak, United States).

### Quantitative Real-Time Polymerase Chain Reaction (qRT-PCR) Analysis

TRI Reagent (Molecular Research Center, United States) was used to isolate total RNA from mouse brain tissue. 1 μg of RNA was reverse−transcribed to complementary DNA with RevertAid First Strand cDNA Synthesis Kit (Thermo Fisher Scientific, United States). Primer sequences used for qRT-PCR were as follows: mFAM19A5-F, 5′-AGG TGA ATG ACC CCC TTC GT-3′; mFAM19A5-R, 5′-TGA CTC TGC TCC CCA GCT TC-3′; mGAPDH-F, 5′-ATC CTG CAC CAC CAA CTG CT-3′; mGAPDH-R, 5′-GGG CCA TCC ACA GTC TTC TG-3′. Real−time polymerase chain reaction was performed on CFX96 Touch^TM^ Real-Time PCR detection system using the SsoAdvanced Universal SYBR^®^ Green Supermix (Bio-Rad, United States). Gene expression was normalized to GAPDH level, and the relative quantity of mRNAs was calculated based on the comparative Cq method.

### Quantification and Statistical Analysis

To quantify the cytoplasmic and nuclear X-gal precipitates of the naïve and TBI-induced brain, the acquired confocal images from the cortex and corpus callosum were converted to 3D images using IMARIS software (IMARIS9.0, Bitplane AG, Zurich, Switzerland). The “Surface tool” of the IMARIS software was used to mark all the signals detected in the X-gal fluorescence channel, and the total X-gal surface was set according to the result. Then, the “Coloc tool” was used to filter and generate the channel indicating the overlap regions of the Hoechst and X-gal signals. The cytoplasmic X-gal surface was calculated as the total X-gal surface minus the “Coloc” surface, and the nuclear X-gal was calculated as the surface areas of the total X-gal surface minus the cytoplasmic X-gal surface. Then the number and the volume of the cytoplasmic and nuclear surfaces were calculated. The number and volume of the X-gal signal were acquired from the central part of the brain tissue section images, which included the injury lesion sites.

The images were taken of three mice from each group (naïve and TBI). For qRT-PCR, total RNA was extracted from 9 naïve and 12 TBI-induced mice brains. All statistical analyses were performed using GraphPad Prism 5 software (GraphPad software, Inc., La Jolla, CA). Data are shown as the means ± standard errors of the mean. For multiple comparisons, one-way ANOVA was performed, followed by Newman-Keuls multiple comparisons test. The criterion for statistical significance was set at a p value less than 0.05.

## Results

### FAM19A5 Expression During Embryogenesis and in the Postnatal Mouse Brain

To understand the functional mechanism of FAM19A5 in the brain, we first investigated the expression pattern of FAM19A5 in the developing mouse brain. To assess this pattern, X-gal staining was employed on the brain tissue of *FAM19A5-LacZ* KI mice. *FAM19A5-LacZ* KI homozygote mice exhibited stronger X-gal staining than heterozygote mice, and wild-type littermates did not generate blue precipitations (Supplementary Figure 1C). These results were further corroborated by those of the *in situ* hybridization assay (Supplementary Figure 2).

The whole mount X-gal staining at embryonic stages E10.5, E12.5, and E14.5 demonstrated predominant expression of FAM19A5 in the brain and spinal cord (Figure 1B). X-gal signal was observed from E10.5. The sagittal sections of E14.5 displayed positive X-gal staining in the brain, spinal cord, and dorsal root ganglion, which is consistent with the *in situ* hybridization result showing expression of FAM19A5 at the mRNA level (Figure 1C). Both X-gal staining (Figure 1D) and the *in situ* hybridization technique (Supplementary Figure 2A) showed FAM19A5 expression in the ventricular zone as well as marginal zone during the neurogenesis period, including at E12.5, E14.5, and E16.5. X-gal staining was also observed in the ganglionic eminence at E12.5, but this staining became weaker at E14.5 and E16.5. FAM19A5 expression was maintained during the late neurogenesis stage (E18.5) in the ventricular zone and subventricular zone. Furthermore, the coronal section views at postnatal stages P4.5, P10.5, P15.5, and P20.5 revealed the broad expression pattern of FAM19A5 in the brain, including the olfactory bulb, corpus callosum, piriform cortex, caudate putamen, hippocampus, amygdala, thalamus, entorhinal cortex, and cerebellum (Figure 1E). These results are consistent with those of the *in situ* hybridization assay (Supplementary Figure 2A), showing continuous whole brain expression of FAM19A5 from the early embryonic stages to the postnatal periods.

### Characterization of Cell Types Expressing FAM19A5 in the Adult Brain

**FIGURE 2:**
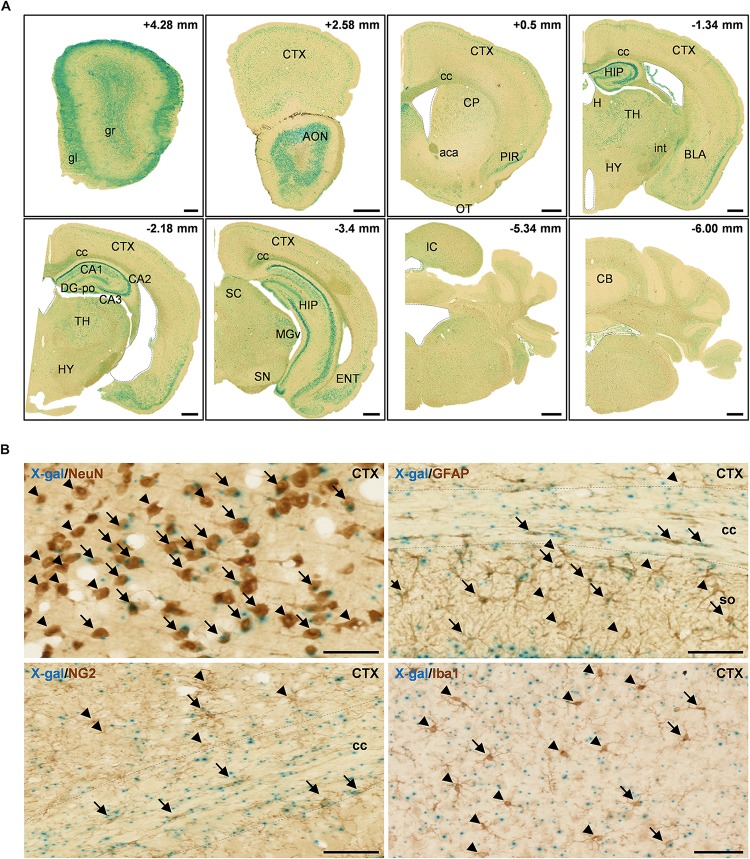
X-gal staining of the adult *FAM19A5-LacZ* knock-in (+/+) mouse brain. **(A)** X-gal staining of coronal sections of 10-week old adult male mice. The section’s distance from the Bregma point is indicated at the upper right hand corner of each box. The lateral ventricle, third ventricle, fourth ventricle, and aqueduct of Sylvia are demarcated by dashed lines. **(B)** X-gal staining combined with 3, 3′-diaminobenzidine staining on adult mouse brain sections. Brain sections incubated in X-gal staining solution for 24 h were immune-labeled for various cellular markers: NeuN for neuron, GFAP for astrocyte, NG2 for oligodendrocyte precursor cell, and Iba1 for microglia. X-gal signals (blue) are seen in subpopulations of NeuN^+^, GFAP^+^, NG2^+^, and Iba1^+^ cells in the adult mouse brain. Arrows and arrow heads depict cell marker positive cells with and without X-gal signals, respectively. The areas for the corpus callosum are indicated by dashed lines. aca, anterior commissure, anterior part; AON, anterior olfactory nucleus; BLA, basolateral amygdaloid nucleus, anterior part; CA1, field CA1 of the hippocampus; CA2, field CA2 of the hippocampus; CA3, field CA3 of the hippocampus; CB, cerebellum; cc, corpus callosum; CP, caudate putamen; CTX, cerebral cortex; DG-po, polymorphic layer of the dentate gyrus; ENT, entorhinal cortex; gl, glomerular layer of olfactory bulb; gr, granular layer of olfactory bulb; H, habenula; HIP, hippocampal region; HY, hypothalamus; int, internal capsule; IC, inferior colliculus; MGv, medial geniculate nucleus, ventral part; OT, olfactory tubercle; PIR, piriform cortex; SC, superior colliculus; SN, substantia nigra; so, stratum oriens of the hippocampus; TH, thalamus. Scale bars represent 500 μm in panel **A** and 50 μm in panel **B**.

In adult mice, FAM19A5 was broadly expressed in many regions of the brain. However, relatively high-intensity X-gal signals were found in several brain areas, such as the olfactory bulb, anterior olfactory nucleus, piriform cortex, hippocampus, thalamus, amygdala, entorhinal cortex, superior colliculus, and inferior colliculus (Figure 2A). In the cerebral cortex, X-gal precipitates were broadly distributed in layers 2/3 and 5 where pyramidal neurons are enriched; X-gal signals were also found in some neurons in layer 4. In the hippocampus, pyramidal neurons in the CA regions (CA1, CA2 and CA3) displayed the presence of X-gal signals. However, the granular neurons of the dentate gyrus hardly exhibited X-gal precipitation (Figure 2A and Supplementary Figure 1C). Besides, brain regions with white matter such as the corpus callosum, anterior commissure, and internal capsule also showed X-gal precipitation (Figure 2A).

FAM19A5 appears to be expressed not only in neurons but also in glial cells, as these cells are widely spread across the white matter along with the axon bundles (Figure 2A and Supplementary Figure 1C). To explore the identity of cells expressing FAM19A5, X-gal–stained sections were further immunostained with various brain cell-type markers: NeuN for neurons, GFAP for astrocytes, NG2 for OPCs, and Iba1 for microglia. X-gal and DAB double staining displayed FAM19A5 expression in the subpopulations of all cell types, including neurons, astrocytes, OPCs, and microglia (Figure 2B). In particular, a large portion of NeuN^+^ cells in the cortical regions and NG2^+^ cells in the corpus callosum were double stained with X-gal.

**FIGURE 3:**
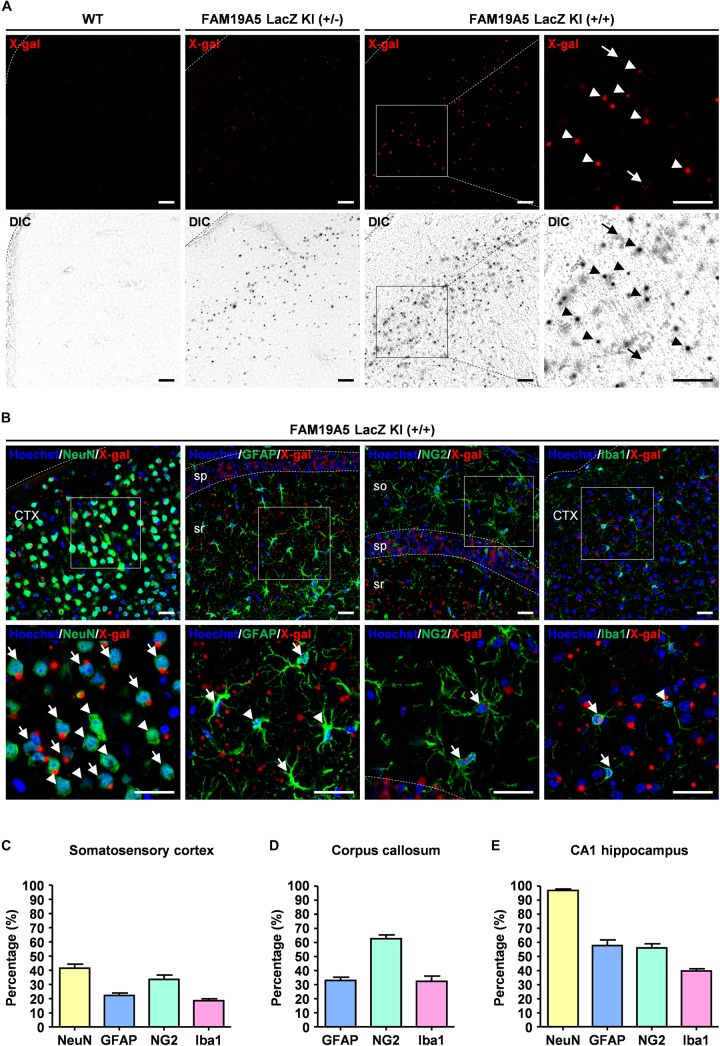
Quantification of X-gal^+^ neuronal and glial cells via direct confocal acquisition of X-gal fluorescence. **(A)** Confocal acquisition of fluorescence from X-gal precipitates in the wild-type (WT) and *FAM19A5-LacZ* knock-in (KI) mouse brains. Fluorescence signals were stronger in *FAM19A5-LacZ* KI homozygote (+/+) mice than in heterozygote (+/−) mice. However, these fluorescence signals were not observed in the WT mouse brains. Differential interference contrast (DIC) images were used to determine whether the florescence signal originated from X-gal precipitates. Arrow heads indicate florescence from X-gal precipitates but arrows represent auto-fluorescence signals that are not driven from X-gal precipitates. **(B)** X-gal staining combined with immunofluorescence staining for cell type-specific markers of the adult *FAM19A5-LacZ* KI (+/+) mouse brain. Brain sections incubated for 24 h in X-gal solution were immunostained for cell markers, including NeuN, GFAP, NG2, and Iba1. X-gal fluorescence signals were observed in subpopulations of neurons (NeuN), astrocytes (GFAP), oligodendrocyte precursor cells (NG2), and microglia (Iba1). Arrows and arrow heads represent cell-type marker^+^ cells with and without X-gal fluorescence signal, respectively. **(C–E)** Quantification of X-gal^+^ cells in the somatosensory cortex **(C)**, medial corpus callosum **(D)**, and CA1 region of the hippocampus **(E)**. Number of X-gal^+^ cells out of cell-type marker^+^ cells were counted based on both fluorescence and DIC images. Data were presented as the means ± standard errors of the mean (*n* = 5). CTX, cerebral cortex; so, stratum oriens of the hippocampus; sp. pyramidal cell layer of the hippocampus; sr, stratum radiatum of the hippocampus. Scale bar represents 50 μm.

To quantify the portion of X-gal^+^ cells out of the total population of each cell type, direct confocal fluorescence acquisition technique was applied to the X-gal staining (Levitsky et al., 2013). Fluorescence dots were observed in both *FAM19A5-LacZ* KI (+/−) and *FAM19A5-LacZ* KI (+/+) mice but not in wild-type mice. The fluorescence dots mostly overlapped with the X-gal signals seen by differential interference contrast microscopy, with few exceptions of autofluorescent signals (Figure 3A). X-gal staining combined with immunofluorescence staining again showed X-gal fluorescence dots in various types of cells, including neurons, astrocytes, OPCs, and microglia (Figure 3B).

Using this technique, the proportion of FAM19A5 expressing cells out of NeuN^+^, GFAP^+^, NG2^+^, and Iba1^+^ cells were quantified in three different brain areas: the somatosensory cortex, corpus callosum, and CA1 region of the hippocampus. In the somatosensory cortex, X-gal precipitations were found in 41% of NeuN^+^, 22% of GFAP^+^, 33% of NG2^+^, and 18% of Iba1^+^ cells (Figure 3C and Supplementary Figure 3A). In the medial corpus callosum, 62% of NG2^+^, 32% of GFAP^+^, and 32% of Iba1^+^ cells exhibited X-gal staining (Figure 3D and Supplementary Figure 3B). In the CA1 region of the hippocampus, most (97%) NeuN^+^ neuronal cells exhibited X-gal^+^, and 30∼50% of glial cells around the CA1 region were co-stained with X-gal (Figure 3E and Supplementary Figure 3C).

### Increased FAM19A5 Expression in Response to Traumatic Brain Injury

**FIGURE 4:**
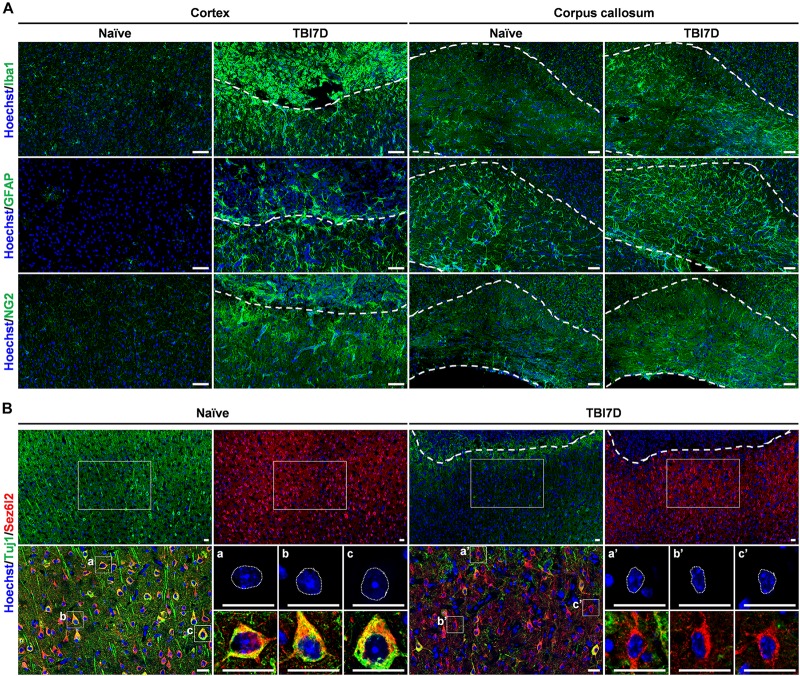
Pathohistological changes of the brain in response to traumatic brain injury. **(A)** Immunostaining for Iba1^+^ microglia/macrophage, GFAP^+^ astrocytes and NG2^+^ oligodendrocyte precursor cells in the cortex and corpus callosum of naïve and TBI-induced brain tissue of *FAM19A5-LacZ* knock-in (+/+) mice at 7 days after TBI (TBI7D). Dashed lines in the cortex of the TBI mouse brain show the border between the injury core (upper part) and penumbra (lower part); dashed lines in the corpus callosum (right panels) indicate their border with the cortex (upper) or with the lateral ventricle (lower). Scale bar, 50 μm. **(B)** Immunostaining for Tuj1^+^ and Sez6l2^+^ neurons in the cortex of naïve and TBI-induced brain tissue. Antibodies for Tuj1 (green) and Sez6l2 (red) were double immunostained and nuclei were stained with Hoechst (blue). Dashed lines in the cortex of the TBI mouse brain show the border between the injury core and penumbra. Magnified images (left bottom of each group) show colocalizations of Tuj1 and Sez6l2 in the neuronal cells. Nuclear morphology of individual neurons was further magnified (right bottom of each group). Sez6l2^+^ neurons in the TBI group were marked by condensed nuclei and decreased Tuj1 signal. Scale bar, 20 μm.

To investigate the transcriptional regulation of the *FAM19A5* gene in a pathological condition, we determined changes in X-gal staining intensity in the TBI-induced brain of *FAM19A5-LacZ* KI mice. Changes in the numbers and morphologies of microglia/macrophages, astrocytes, and OPCs are typical pathohistological characteristics of the injured brain (Shechter and Schwartz, 2013; Burda and Sofroniew, 2014). Therefore, we first examined such pathohistological changes in the TBI-induced brain of *FAM19A5-LacZ* KI mice. TBI significantly induced a large accumulation of Iba1^+^ microglia (or blood-born macrophages) in the injury core and penumbra of the cortex, as well as the corpus callosum (Figure 4A). To further distinguish these changes by cell type, the injured brain was double stained with anti-Iba1 antibody favoring microglia and anti-CD45 antibody preferring blood-born leukocytes. Microglia having strong staining for Iba1 but faint staining for CD45 were primarily distributed in the penumbra but also present in the injury core (Supplementary Figures 4Aa,a’). By contrast, blood-born macrophages with weak Iba1 staining but strong CD45 staining were predominantly present in the injury core along the injury border (Supplementary Figures 4Ab,b’). Round-shaped non-microphage leukocytes were stained by only anti-CD45 antibody. The majority of these cells were found in the injury core but some were also observed in the injury penumbra (Supplementary Figures 4Ac,c’). An increased number of reactive astrocytes labeled with anti-GFAP antibody also gathered along the border between the penumbra and the injury core but primarily were collected in the penumbra (Figure 4A). In addition, the intensity of NG2^+^ signals in OPCs were increased in the penumbra and corpus callosum (Figure 4A). By contrast, the TBI-induced brain exhibited lower signals for myelin basic protein representing fully mature oligodendrocytes than the naïve brain (Supplementary Figure 4B). Neurons were immunostained with two different marker proteins (Yaguchi et al., 2014). Neurons in the penumbra of the TBI-induced mouse brain exhibited lower intensity of Tuj1 immunostaining than those in the cortex of the naïve mouse brain (Figure 4B). In addition, the nuclei of the neurons in the penumbra looked largely shrunken in size. Interestingly, unlike Tuj1, Sez6l2 was relatively well stained in both the TBI-induced and naïve brains with similar intensities (Figure 4B).

**FIGURE 5:**
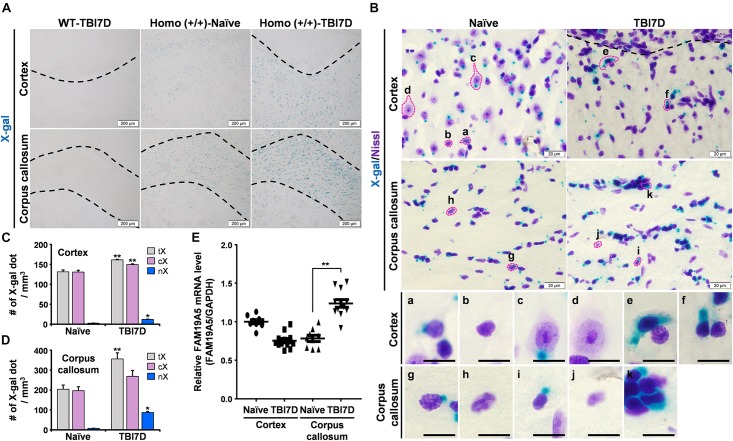
TBI increased X-gal staining in the *FAM19A5-LacZ* knock-in (+/+) mouse brain. **(A)** X-gal staining of the naïve and injured brain at 7 days after TBI (TBI7D). Dashed lines in the cortex of the TBI mouse brain show the border between the injury core and penumbra (left and right top). Areas between dashed lines are the corpus callosum (bottom). **(B)** X-gal and Cresyl violet (Nissl) staining in the cortex and corpus callosum. Images for individual cells were magnified depending on nuclear morphology and X-gal staining pattern (a-k). Scale bar, 10 μm. **(C,D)** Number of X-gal dots in the cortex **(C)** and corpus callosum **(D)**. Number of total X-gal (tX), cytoplasmic X-gal (cX), and nuclear X-gal (nX) was measured from confocal images for fluorescence of X-gal and Hoechst using IMARIS software. Values are the means ± standard errors of the mean. ^∗^*P* < 0.05 and ^∗∗^*P* < 0.01 vs. naïve (*n* = 3). **(E)** qRT-PCR analysis of FAM19A5 mRNA levels in the cortex and corpus callosum of naïve and TBI-induced mice. Values are the means ± standard errors of the mean. ^∗∗^*P* < 0.01 vs. naïve (naïve, *n* = 9; TBI, *n* = 12).

Because TBI increases senescence-associated β-gal activity at pH 6.0 (Tominaga et al., 2019), we performed X-gal staining at pH 7.4 in the TBI-induced brain of the wild-type mice. No X-gal staining was observed in the TBI-induced brain of the wild-type mouse, indicating no senescence-associated β-gal activity in this assay system. Then we compared the X-gal staining pattern between the naïve and TBI-induced brain of *FAM19A5-LacZ* KI mice, revealing increased X-gal staining in the penumbra and corpus callosum of the TBI group (Figure 5A).

To distinguish the cell types with increased X-gal staining in the TBI-induced brain, tissue sections were further stained with Cresyl violet (Nissl) to examine the morphology of X-gal–stained cells (Figure 5B). Nuclear sizes and morphologies are the well-known criteria for distinguishing neurons from non-neuronal glial cells in the brain. In general, neurons have a large nucleus with high euchromatin content, while non-neuronal glial cells contain a small condensed nucleus with heterochromatin (García-Cabezas et al., 2016). A variety of cell types were determined based on the nuclear morphology and X-gal–staining pattern. In the cerebral cortex of naïve mice, some glial cells exhibited a small amount of X-gal precipitate in the cytoplasm (Figure 5B, a) while others showed none (b). Some neurons also showed X-gal precipitate in the cytoplasm (c) but others did not (d). Likewise, some populations of glial cells in the corpus callosum exhibited a weak X-gal staining in the cytoplasm (g). Thus, under the normal condition, some populations of neuronal and non-neuronal cells of *FAM19A5-LacZ* KI mice may express low level of β-gal, giving rise to a single small punctual shaped X-gal precipitate within the cytoplasm outside of nucleus (a and c). In the TBI-induced brain, however, increased X-gal precipitations were observed, expanding to the nucleus and covering a large area of cytoplasm in both neuron-like cells (e and f) in the penumbra of the cortex and glia-like cells in the corpus callosum (k).

We then determined the number of X-gal-precipitated regions in the cytoplasm and nucleus in the cortex and corpus callosum. TBI significantly increased the number of X-gal precipitated regions that entered the nucleus of cells in both the injury penumbra (Figure 5C) and the corpus callosum (Figure 5D). The number of the X-gal dots (which occurred only in the cytoplasm) slightly increased in the TBI group. We examined FAM19A5 mRNA levels using qRT-PCR. The result showed a significant increase in FAM19A5 mRNA levels in the corpus callosum of the TBI mice (Figure 5E). However, FAM19A5 mRNA levels in the cortical area were slightly decreased in the TBI mice compared to that of the wild type mice. This result is likely that the cortical region of the wild type mice containing the layer 2/3 region exhibits a relatively higher level of FAM19A5, while TBI mice largely lost the layer 2/3 region.

### Increased FAM19A5 Expression in the Subpopulation of Neurons and Oligodendrocyte Precursor Cells of the TBI-Induced Brain

**FIGURE 6:**
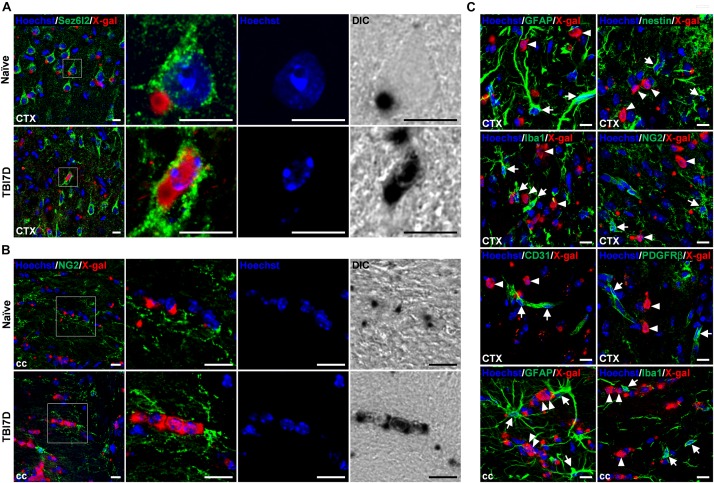
Nuclear X-gal precipitation in Sez6l2^+^ neurons and NG2^+^ oligodendrocyte precursor cells of the TBI-induced brain. **(A)** Fluorescence and differential interference contrast (DIC) images of the cortical region in naïve and TBI7D brain sections after X-gal staining combined with Sez6l2 immunostaining and Hoechst staining. Boxed regions are magnified in the right panels showing nuclear morphology and X-gal staining pattern. **(B)** Fluorescence and DIC images of NG2^+^ OPCs in the corpus callosum. Nuclear X-gal was stained in NG2^+^ OPCs of the TBI-induced group. Boxed regions are magnified in the right panels showing nuclear morphology and X-gal staining pattern. **(C)** Fluorescence images for X-gal staining combined with GFAP, nestin, Iba1, NG2, CD31, or PDGFRβ immunostaining of the penumbra and corpus callosum of TBI7D brain section. Arrows and arrowheads indicate cell type–specific marker^+^/nuclear X-gal^–^ and cell type–specific marker^–^/nuclear X-gal^+^ cells, respectively. Scale bar, 10 μm.

To identify cell types with nuclear X-gal staining by TBI, the brain tissue of *FAM19A5-LacZ* KI mice was further immunostained with various cell type–specific markers. It is important to note that insoluble blue precipitates of X-gal hamper immune reactions, therefore immunostaining could not occur in the X-gal–stained region (Levitsky et al., 2013). Out of many cell-type markers, anti-Sez6l2 antibody, a neuronal cell marker, immunostained cells with nuclear X-gal signals in the penumbra of the TBI-induced brain (Figure 6A), whereas it stained healthy neuronal cells with cytoplasmic X-gal signals in the naïve brain section. The nuclear X-gal^+^ cells in the penumbra region have nuclei that are slightly larger than those of glial cells but smaller than those of normal neuronal cells. In addition, Hoechst staining showed that heterochromatins were primarily located along the nuclear envelope border in the nuclear X-gal^+^ cells, whereas heterochromatins were in the center near nucleoli in normal healthy neurons (Figure 6A). The neuronal markers such as NeuN, Tuj1, and Map2, however, were hardly stained in the cells with nuclear X-gal signals (Supplementary Figures 5A–C). The lack of signals is likely due to decreased expression of these marker proteins under an injury condition. Indeed, our study revealed a drastic decrease in signal intensity for Tuj1 in the penumbra region in the absence of X-gal staining (Figure 4B). Alternatively, the increased X-gal precipitation may interfere with immunostaining when these marker antibodies are used (Levitsky et al., 2013). In addition, the nuclear X-gal^+^ cells were not co-stained with markers for non-neuronal cells, such as GFAP and nestin for astrocytes (Figure 6C and Supplementary Figures 5D,E), Iba1 for microglia/macrophage (Figure 6C and Supplementary Figure 5F), NG2 and Olig2 for oligodendrocytes or their precursor cells (Figure 6C and Supplementary Figures 5G,H), CD31 for endothelial cells (Figure 6C and Supplementary Figure 5I), or PDGFRβ for pericytes (Figure 6C). Furthermore, nuclear X-gal^+^ cells located in the injury penumbra were not stained with Ki67 for a cell proliferation marker (Supplementary Figure 5J) or TUNEL/active caspase-3 for cell death markers (Supplementary Figure 5K).

We were able to see a large portion of GFAP^+^ astrocytes in the injury core were cytoplasmic X-gal^+^, albeit no nuclear X-gal staining (Supplementary Figure 6A). In the injury core, some of NG2^+^ and PDGFRβ^+^ pericytes, and CD31^+^ endothelial cells were cytoplasmic X-gal^+^ (Supplementary Figures 6B–D). However, we were unable to observe CD45^+^/Iba1^–^ cells with cytoplasmic X-gal staining (Supplementary Figure 6E).

In contrast to the penumbra region, some nuclear X-gal^+^ cells in the corpus callosum of the TBI-induced brain were co-stained with NG2^+^ OPCs (Figure 6B) and O4^+^ postmitotic oligodendrocytes (Supplementary Figure 7A). However, antibodies for Sez6l2 (Supplementary Figure 7B), GFAP (Figure 6C and Supplementary Figure 7C), and Iba1 (Figure 6C and Supplementary Figure 7D) failed to double stain the cells with the nuclear X-gal signal.

## Discussion

Using *FAM19A5-LacZ* KI mice, we demonstrated that the *FAM19A5* gene is primarily expressed in the CNS from an early stage (E10.5) of embryogenesis. In particular, X-gal staining of embryo brains indicates that FAM19A5 is expressed in germinal zones such as the ventricular zone and ganglionic eminence, suggesting a role of FAM19A5 in proliferation and differentiation of neuroglial progenitor cells. Development of the CNS starts with the formation of the neural tube from the neural plate (Sakai, 1989). The neuroepithelial cell layer adjacent to the ventricle, named the ventricular zone, is the pool for neural stem cells (NSCs) that are capable of self-renewal and differentiate into various types of brain cells including neurons and glial cells to form an organized and functional brain. In rodents, the corticogenesis period ranges from E10.5 to E18.5, followed by the gliogenesis period which completes after birth (Götz and Huttner, 2005; Molyneaux et al., 2007; Agirman et al., 2017). X-gal staining at E12.5 also shows FAM19A5 expression in the ganglionic eminence, the place for the first emergence of OPCs that can differentiate into either oligodendrocytes or astrocytes depending on context (Ffrench-Constant and Raff, 1986). In addition, progenitor cells for GABAergic interneurons in the ganglionic eminence at E12.5 migrate tangentially to the neocortex and occupy layer 4 (Anderson et al., 1997).

In the adult brain, X-gal staining combined with immunostaining for cell-type markers suggests that FAM19A5 is expressed in diverse cell types, including neurons, astrocytes, OPC, and microglia. Expression of FAM19A5 in diverse cell types is consistent with the results of single cell RNA-sequencing obtained from mouse and human brains (Zhang et al., 2014, 2016). It is noteworthy that FAM19A5 is expressed only in the subpopulations of these cell types. For instance, FAM19A5 is broadly expressed in pyramidal neurons in layers 2/3 and 5 of the cortex and in the CA regions of the hippocampus, but not in granular neurons in the dentate gyrus of the hippocampus. In addition, more than 62% of NG2^+^ OPCs in the corpus callosum are X-gal^+^ but only 30% of NG2^+^ OPCs in the cortex are X-gal^+^. This restricted expression of FAM19A5 in diverse cell types is likely due to the heterogeneity of NSCs (Alvarez-Buylla et al., 2008; Agirman et al., 2017).

It is well established that the presence of various types of NSCs are morphologically and transcriptionally distinct, including neuroepithelial cells, radial glial cells (RGCs), intermediate progenitor cells, short neural precursor cells, and outer RGCs (oRGCs). They give rise to the distinct subtypes of neurons and glial cells in a time-scaled manner (McCarthy et al., 2018). Furthermore, such differentiation of NSCs is regulated by multiple intrinsic and extrinsic cues including sonic hedgehog (Shh), Wnt/β-catenin, bone morphogenic proteins, and fibroblast growth factors (Li et al., 1998; Morrow et al., 2001; Panchision et al., 2001; Hirabayashi et al., 2004; Shen et al., 2006; Kang et al., 2009; Xu et al., 2010; Draganova et al., 2015). However, the reason for FAM19A5 expression in pyramidal neurons but not in granular neurons of the dentate gyrus is unclear and needs to be further investigated.

In the mouse forebrain, OPCs emerge first from the medial ganglionic eminence at E12.5, second from the lateral ganglionic eminence at E15.5 (Tekki-Kessaris et al., 2001; Chapman et al., 2013), and third from the subventricular zone around birth (Kessaris et al., 2006). In particular, OPCs from the subventricular zone migrate locally to populate the corpus callosum, as well as cortical areas (Kessaris et al., 2006). Therefore, OPCs originate from multilineage competent neuroepithelial precursors in a stereotypic fashion during mouse embryogenesis (Goldman and Kuypers, 2015). High levels of X-gal staining in the medial ganglionic eminence at E12.5 but lower levels of X-gal staining in lateral ganglionic eminence at E16.5 may account for the heterogeneity of FAM19A5-expressing cells among OPC populations. Furthermore, enriched X-gal^+^ OPCs in the corpus callosum may suggest the large portion of FAM19A5-expressing OPCs in the corpus callosum originate from the subventricular zone around birth.

The present study also showed substantial X-gal staining in the subpopulation of astrocytes, even though the proportion of X-gal^+^ astrocyte differ depending on brain regions. Astrogenesis has been known to start after completion of the neurogenic period and before generation of oligodendrocytes (Malatesta et al., 2000). However, a recent report provided evidence for the appearance of first astrocyte around E16 (Bayraktar et al., 2015), increasing abruptly after cessation of neurogenesis around E18.5 and continuing postnatally (Qian et al., 2000; Molyneaux et al., 2007; Ge et al., 2012). Thus, X-gal staining in the germinal zone at E18.5 may suggest roles of FAM19A5 in gliogenesis from NSCs. In addition, astrocytes can arise from OPCs (Ffrench-Constant and Raff, 1986; Zhu et al., 2008; Suzuki et al., 2017). OPCs can give rise to both oligodendrocytes and astrocytes, which are modulated by extracellular signals temporospatially (Zhu et al., 2008; Tanner et al., 2011).

Unlike neurons, astrocytes, and oligodendrocytes, microglia arise during the first wave of yolk sac hematopoiesis at E7.5, which occupies the brain around E9.5 (Ginhoux et al., 2010; Gomez Perdiguero et al., 2015; Sheng et al., 2015). However, a study by De et al. (2018) showed the presence of two subpopulations of microglia, non-Hoxb8 and Hoxb8 microglia. The non-Hoxb8 microglia are generated from the first wave while the Hoxb8 microglia are from the second wave of yolk sac hematopoiesis, which can be detected in the brain only from E12.5.

One interesting observation of the present study is that TBI leads to increased FAM19A5 expression in a subset of neuronal populations in the injury penumbra of the cortex and some OPCs in the corpus callosum. TBI induces morphological and functional changes in cells surrounding lesion sites, in addition to recruiting reactive astrocytes, OPCs, microglia, blood-born macrophages, and leukocytes (Werner and Engelhard, 2007). In particular, neurons in the injury penumbra undergo dramatic nuclear condensation with increased heterochromatin formation (Figure 4B) accompanied by decreased expression of NeuN and Tuj1, two typical neuronal marker proteins (Kim et al., 2016). Importantly, neurons with a condensed nucleus exhibited increased X-gal staining that invaded the area of the nucleus. Nuclear X-gal staining is quite unusual because the *LacZ gene*-driven β-gal protein is relatively heavy (116 kDa in molecular weight) and cannot cross over the nuclear envelope under normal conditions (Strasser et al., 2012). Therefore, disruption of the nuclear pore complex (NPC), which is specialized in transporting cellular components larger than 40 kDa, may be responsible for the diffusive activity into the nucleus (Kotwaliwale and Dernburg, 2009). Cellular stress or aging is known to cause nuclear leakage by deterioration of the nuclear pore complex, which would allow cytoplasmic proteins larger than 70 kDa to enter the nucleus (D’Angelo et al., 2009; Fichtman and Harel, 2014). Thus, it can be postulated that neurons under severe stress conditions produce more FAM19A5 promoter–driven β-gal proteins that cross over the nuclear envelope due to nuclear leakage. Because the neurons in the injury penumbra exhibited nuclear condensation and presumptive nuclear envelope leakage, we further examined whether these neurons entered the cell death process using TUNEL and active caspase-3 immunostaining (Toné et al., 2007; Strasser et al., 2012). However, the neurons with nuclear X-gal staining were neither TUNEL^+^ nor active caspase-3^+^, indicating that these neurons were not destined to die yet.

TBI also induced increased X-gal staining in the OPCs of the corpus callosum, even though this place was distant from the lesion site. The neurons in the cortex project axons to a variety of brain regions, including the thalamus and cortex in the other hemisphere, through the corpus callosum where extensive myelination occurs (Tau and Peterson, 2010). TBI is known to cause demyelination of the axons projecting through the white matter of the brain, resulting in axonal degeneration and death of both neurons and oligodendrocytes (Flygt et al., 2013; Dent et al., 2015). After the loss of mature oligodendrocytes by axon degeneration following brain injury, OPCs are increased in number, are activated in response to the changes of the microenvironment, and differentiate to mature oligodendrocytes (Flygt et al., 2016; Bonfanti et al., 2017). The *FAM19A5* promoter–driven X-gal signals were elevated in the NG2^+^ OPCs and O4^+^ postmitotic oligodendrocytes in the corpus callosum. Thus, elevated FAM19A5 expression is likely to be involved in OPC-mediated repair process machinery against injury (Ohtomo et al., 2018).

In summary, the FAM19A5 expression pattern revealed by X-gal staining during mouse embryogenesis and in the adult brain following TBI suggests FAM19A5 is a key regulator in both CNS development and the injury response of the brain. Understanding the function of FAM19A5 in the brain is of particular importance because recent clinical studies have revealed the genetic association of *FAM19A5* with brain development-related symptoms, such as ADHD and autism (Kashevarova et al., 2018), and degenerative disease, such as Alzheimer disease (Herold et al., 2016; Mez et al., 2017). FAM19A5 is expressed in a variety of brain cell types but primarily in pyramidal neurons in the cortex and hippocampus and in OPCs in the corpus callosum, as well as in astrocytes and microglia. Increased expression of FAM19A5 in response to TBI suggests a presumptive role of FAM19A5 in the wound healing process of the brain after injury.

## Data Availability

All the data generated for this study are included in the manuscript and/or the Supplementary Files. The raw data supporting this manuscript will be available from the authors by reasonable request without undue reservation.

## Ethics Statement

This study was carried out in accordance with the recommendations of the Institutional Animal Care and Use Committee of Korea University (KOREA-2016-0091-C3). The protocol was approved by the Institutional Animal Care and Use Committee of Korea University.

## Author Contributions

AS and EC performed the experiments, analyzed the results, and wrote the manuscript. HY, IJ, and HK performed the experiments and analyzed the results. JL designed the experiments and provided the analysis methods. WL wrote the draft of the manuscript. H-CP, HK, and J-IH provided the study conception and performed the data analysis. BK, WK, and JS provided the study conception, designed the experiments, and wrote the manuscript. All authors read the manuscript, contributed to the revisions, and approved its submitted version.

## Conflict of Interest Statement

EC, JL, WK, HK, and BK were employed by the Neuracle Science, Co., Ltd.

The remaining authors declare that the research was conducted in the absence of any commercial or financial relationships that could be construed as a potential conflict of interest.
