# Supplementary material for: FAM19A5 Expression During Embryogenesis and in the Adult Traumatic Brain of FAM19A5-LacZ Knock-in Mice
Source: Front Neurosci. 2019 Aug 30;13:917. doi: 10.3389/fnins.2019.00917 (PMC6730007; doi:10.3389/fnins.2019.00917)
Supplement: Supplementary file 1 [file Data_Sheet_1.pdf]

Supplementary Material

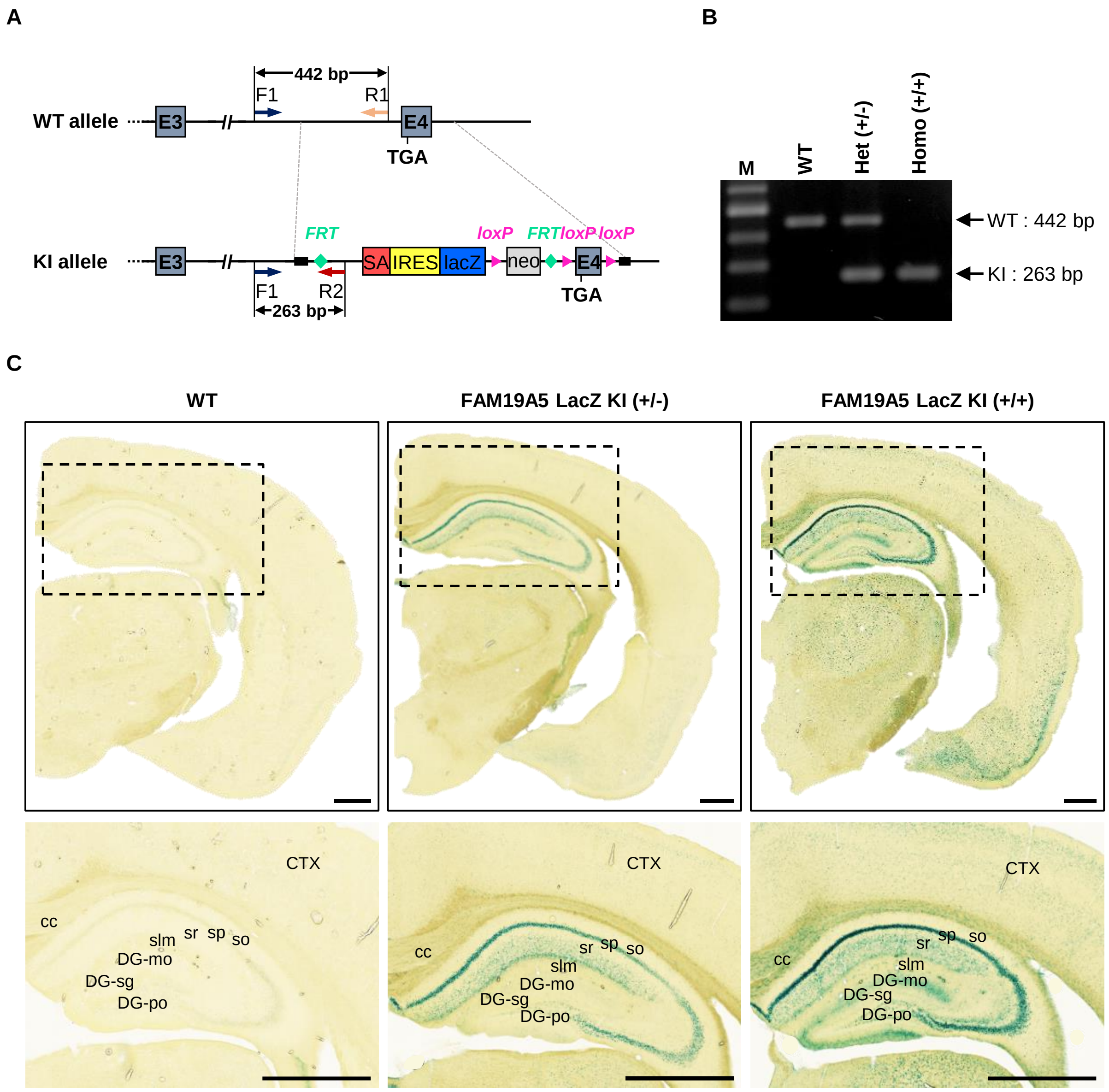

Supplementary Figure 1. X-gal staining of adult *FAM19A5-LacZ* knock-in (KI) mice brain. (A) Schematic diagram showing primers annealing site in the wild-type (WT) allele and *FAM19A5-LacZ* KI allele. Forward primer (F1) and reverse primer (R1) for the WT allele, as well as forward primer (F1) and reverse primer (R2) for KI allele, were used for genotyping mice, producing PCR products with 442 bp and 263 bp, respectively. **(B)** Different genotypes of animals used for X-gal staining: wild type [WT (-/-)], heterozygote of *FAM19A5-LacZ* KI [Het (+/-)] and homozygote of *FAM19A5-LacZ* KI [Homo (+/+)]. **(C)** X-gal staining of adult *FAM19A5-LacZ* KI mice brain and wild-type (WT) littermate mice brain. Coronal brain sections of all three genotypes were subjected to X-gal staining for 48 h. Blue X-gal precipitates were observed in both heterozygote and homozygote of *FAM19A5-LacZ* KI mice brain with negligible signal in wild-type mice brain. The X-gal signals are stronger in *FAM19A5-LacZ* KI (+/+) than *FAM19A5-LacZ* KI (+/-). cc, corpus callosum; CTX, cerebral cortex; DG-mo, molecular layer of the dentate gyrus; DG-po, polymorph layer of the dentate gyrus; DG-sg, granular layer of the hippocampus; slm, lacunosum moleculare layer of the hippocampus; so, oriens layer of the hippocampus; sp, pyramidal cell layer of the hippocampus; sr, stratum radiatum of the hippocampus. Scale bar represents 1000 µm.

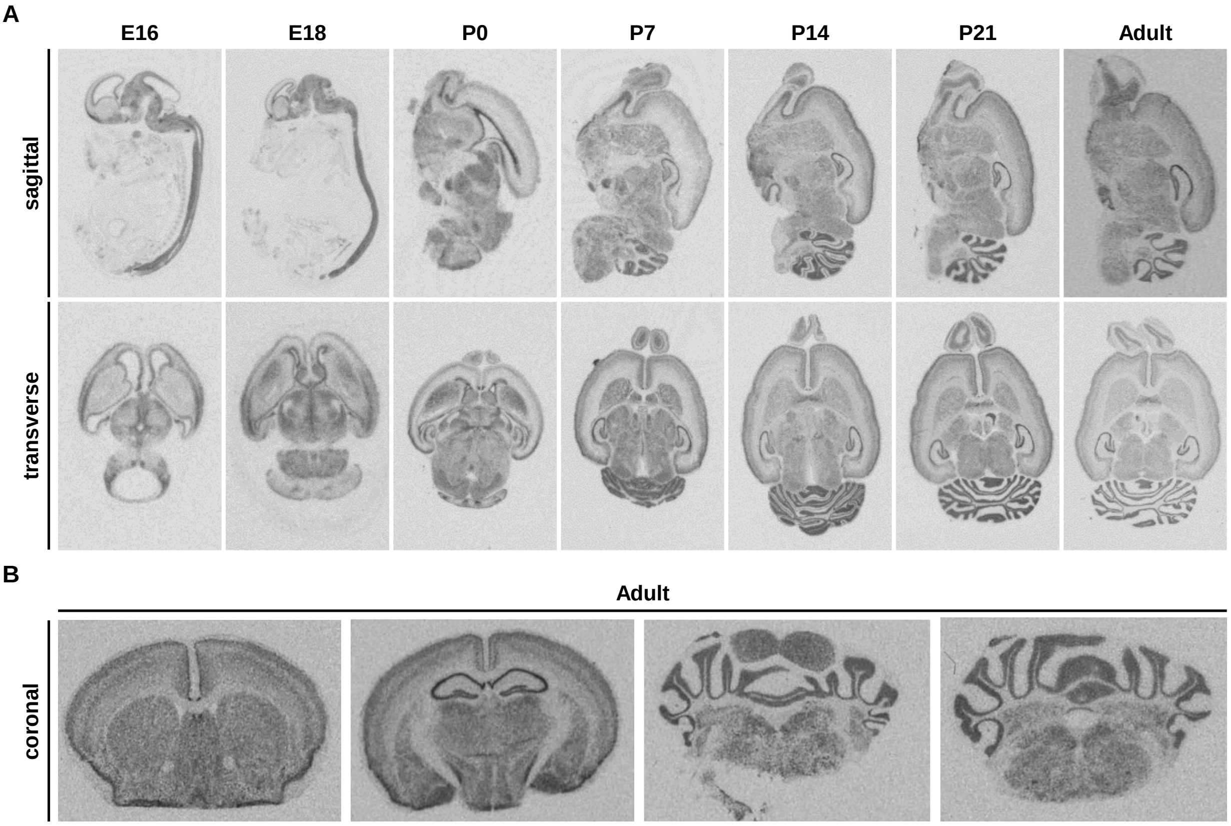

Supplementary Figure 2. In situ hybridization of FAM19A5 in rat and mice brains. (A) Sagittal and transverse views of rat embryo at embryonic days E16 and E18, as well as rat brain at postnatal stages P0, P7, P14, P21, and adult stage. **(B)** Coronal view of the adult mouse brain.

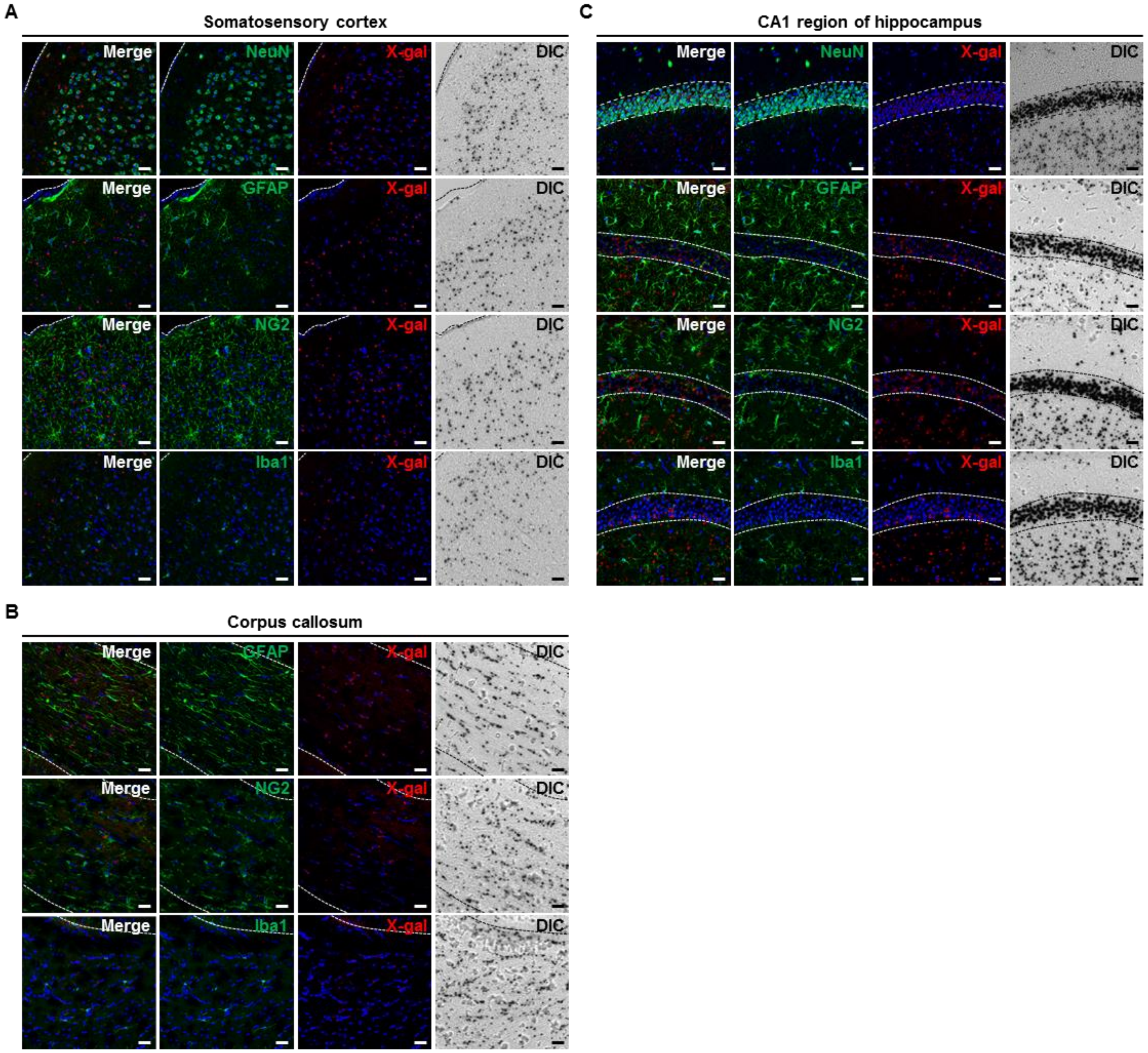

Supplementary Figure 3. X-gal staining combined with immunofluorescence staining in adult *FAM19A5-LacZ* KI (+/+) mouse brain. Brain sections incubated for 24 h in X-gal solution were immunostained for cell markers like NeuN, GFAP, NG2, and Iba1, and imaging was performed in three different brain areas: somatosensory cortex **(A)**, corpus callosum **(B)** and CA1 region of hippocampus **(C)**. X-gal fluorescence signals were observed in subpopulation of neurons (NeuN), astrocytes (GFAP), oligodendrocyte precursor cells (NG2), and microglia (Iba1). Dashed lines in **(A)**, **(B)**, and **(C)** indicate cortical border, corpus callosum border, and CA1 pyramidal cell layer of hippocampus, respectively. Scale bar represents 50 µm.

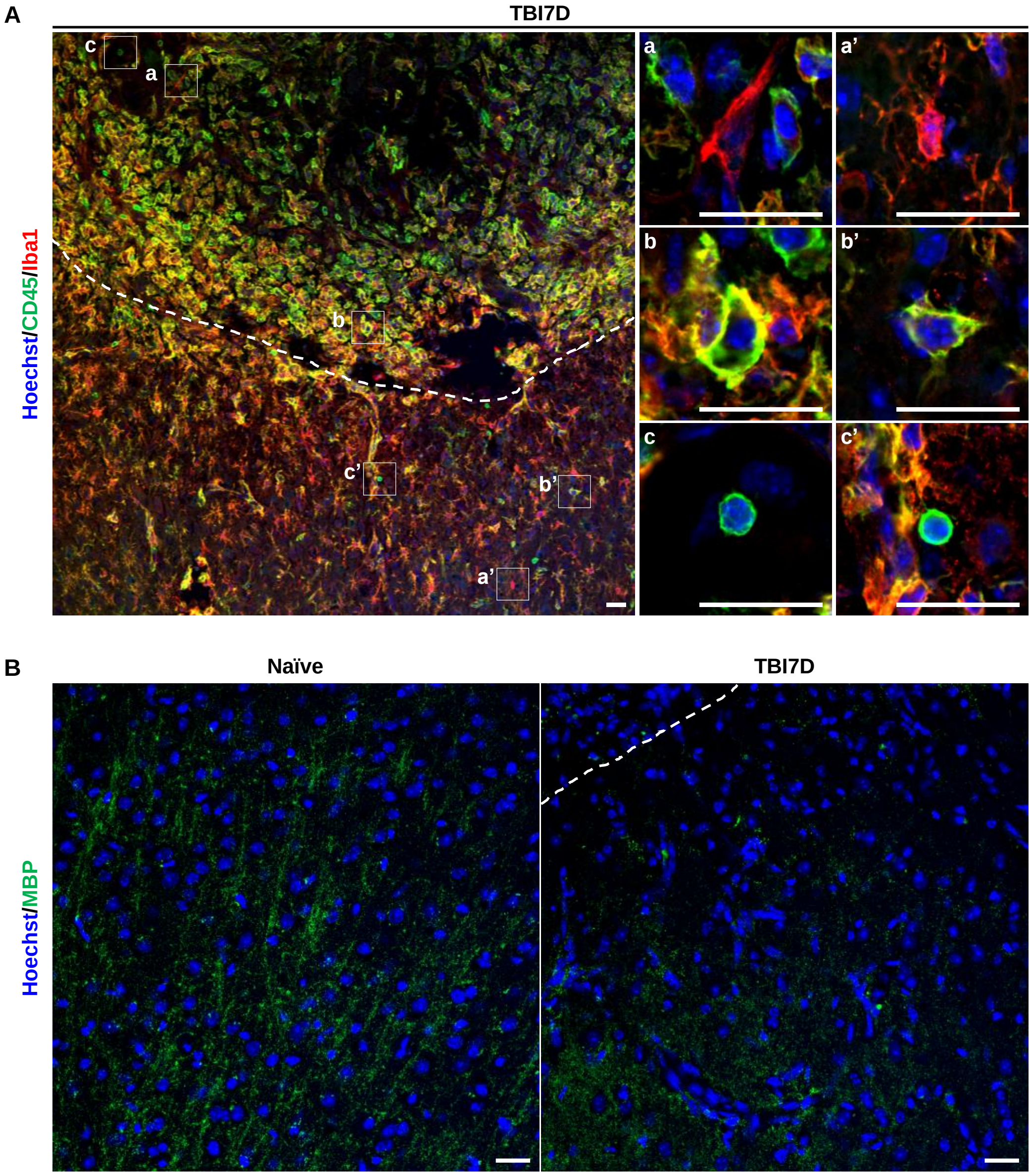

Supplementary Figure 4. Pathohistological changes of the brain after traumatic brain injury. (A) Immunostaining of CD45 (green) and Iba1 (red) in the cortex of TBI-induced brain tissue of FAM19A5 LacZ KI (+/+) mice at 7 days after TBI (TBI7D). Dashed lines in the cortex of the TBI-induced mouse brain show the border between injury core and penumbra. Image for individual cells were magnified depending on the immunoreactivity for CD45 and Iba1 (right). **(B)** Immunostaining for MBP^+ mature oligodendrocytes in the cortex of naive and TBI-induced brain tissue. Dashed lines in the cortex of the TBI-induced mouse brain show the border between injury core and penumbra. Scale bar, 20 µm.

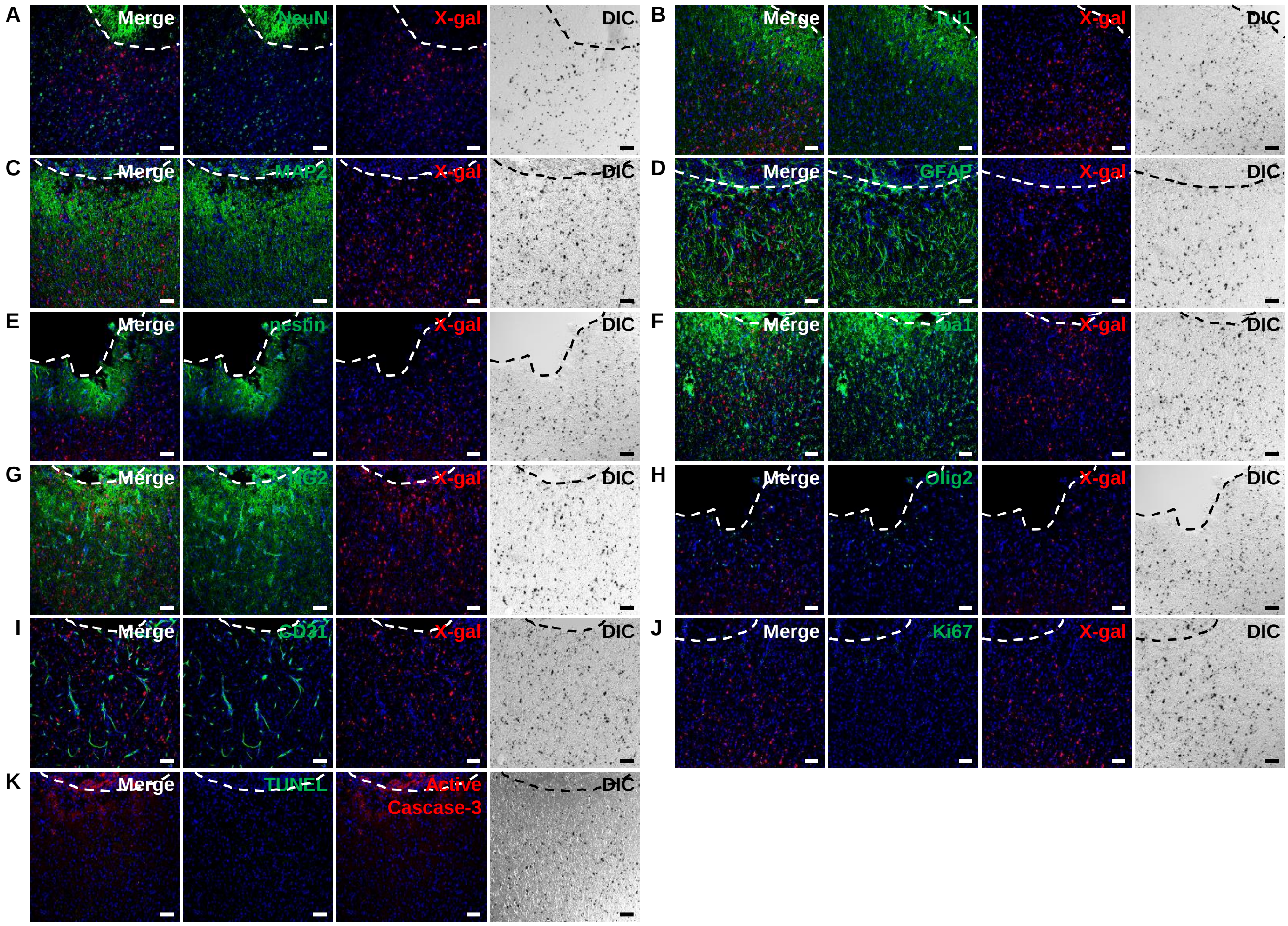

Supplementary Figure 5. X-gal staining and immunostaining of the injury penumbra following traumatic brain injury. X-gal–stained brains were further immunostained with antibodies for NeuN **(A)**, Tuj1 **(B)**, MAP2 **(C)**, GFAP **(D)**, nestin **(E)**, Iba1 **(F)**, NG2 **(G)**, Olig2 **(H)**, CD31 **(I)**, Ki67 **(J)**, TUNEL **(K)**, and active caspase-3 **(K)**. Nuclei were staining with Hoechst. Differential interference contrast (DIC) images were presented. Dashed lines indicate the lesion border. Scale bar represents 50 μm.

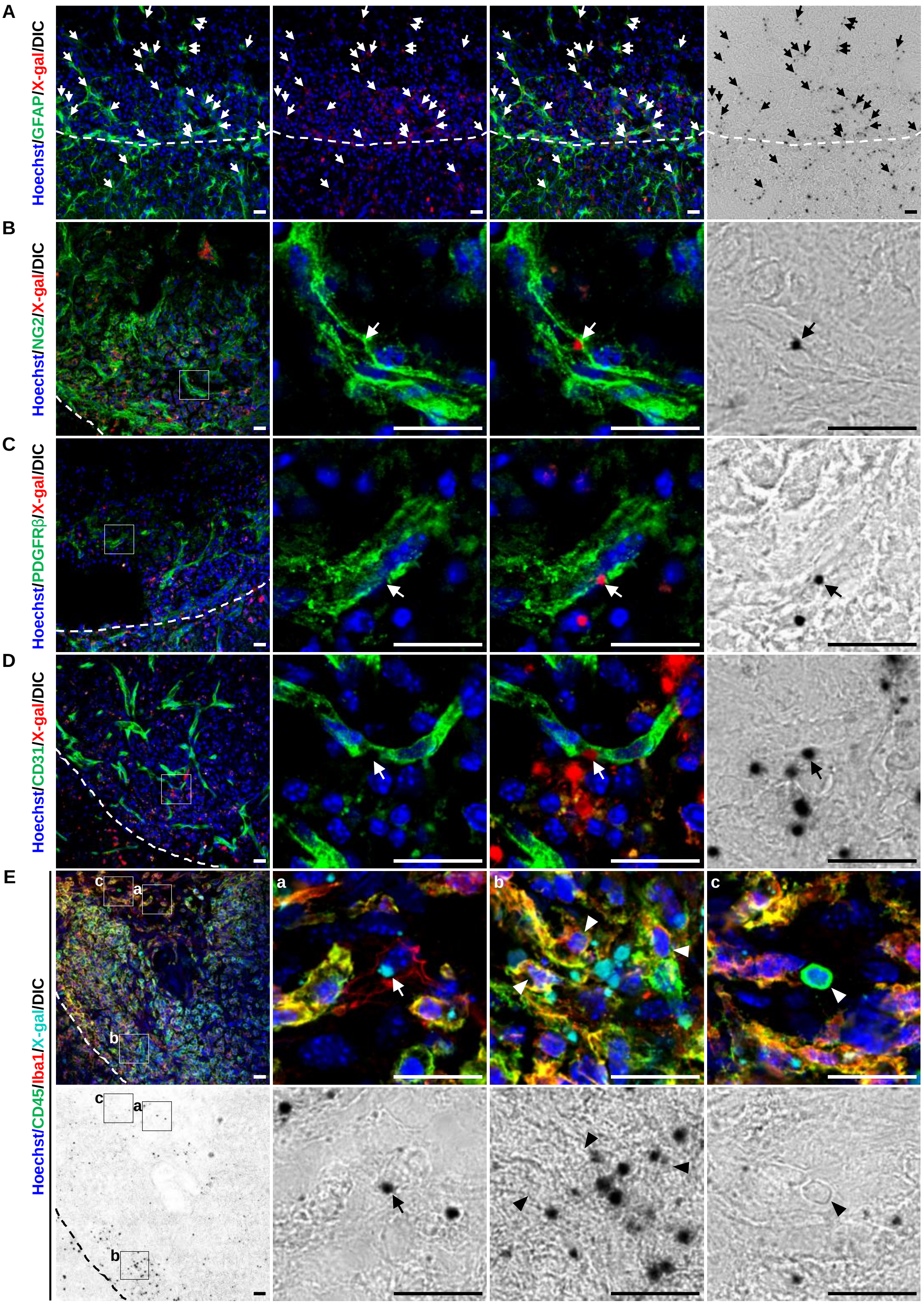

Supplementary Figure 6. X-gal staining and immunostaining of the injury core following traumatic brain injury. (A) X-gal stained brains were immunostained with antibody against GFAP (astrocytes). Arrows indicate cytoplasmic X-gal$^+$/GFAP$^+$ astrocytes. **(B-D)** X-gal stained brains were immunostained with antibodies against NG2 (oligodendrocyte precursor cells, **B**), PDGFRβ (pericytes, **C**), and CD31 (endothelial cells, **D**). Boxed regions are magnified in the right panels showing cellular morphology and X-gal staining pattern. Arrows indicate cytoplasmic X-gal$^+$/cell type-specific marker$^+$ cells. **(E)** X-gal stained brains were immunostained with antibodies against CD45 and Iba1. Boxed regions are magnified in the right panels depending on the immunoreactivity for CD45 and Iba1. Arrows and arrowheads indicate cytoplasmic X-gal$^+$ and cytoplasmic X-gal$^-$ cells, respectively. Nuclei were staining with Hoechst. Differential interference contrast (DIC) images were presented. Dashed lines indicate the lesion border. Scale bar represents 20 μm.

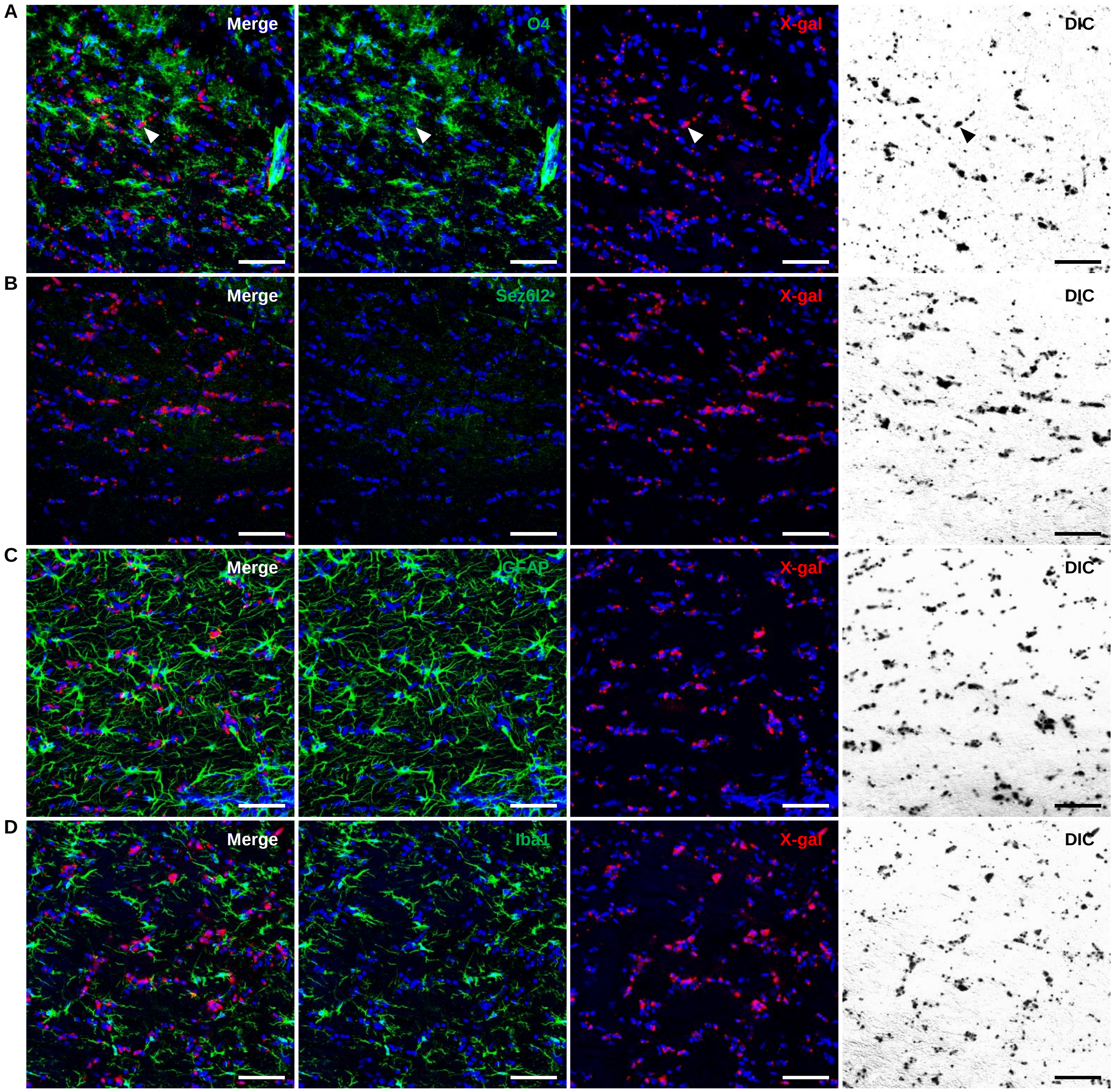

Supplementary Figure 7. X-gal staining and immunostaining of the corpus callosum following traumatic brain injury. X-gal–stained brains were immunostained with antibodies against O4 (oligodendrocyte lineage cells, **A**), Sez6l2 (neurons, **B**), GFAP (astrocytes, **C**), and Iba1 (microglia/macrophage, **D**). Nuclei were staining with Hoechst. Differential interference contrast (DIC) images are presented. Scale bar represents 50 µm.
